# White Matter Connectome Correlates of Auditory Over-Responsivity: Edge Density Imaging and Machine-Learning Classifiers

**DOI:** 10.3389/fnint.2019.00010

**Published:** 2019-03-29

**Authors:** Seyedmehdi Payabvash, Eva M. Palacios, Julia P. Owen, Maxwell B. Wang, Teresa Tavassoli, Molly Gerdes, Annie Brandes-Aitken, Pratik Mukherjee, Elysa J. Marco

**Affiliations:** ^1^Department of Radiology and Biomedical Imaging, Yale School of Medicine, New Haven, CT, United States; ^2^Department of Radiology and Biomedical Imaging, University of California, San Francisco, San Francisco, CA, United States; ^3^Department of Radiology, University of Washington, Seattle, WA, United States; ^4^University of Pittsburg School of Medicine, Pittsburgh, PA, United States; ^5^Department of Psychology and Clinical Sciences, University of Reading, Reading, United Kingdom; ^6^Department of Neurology, University of California, San Francisco, San Francisco, CA, United States; ^7^Department of Applied Psychology, New York University, New York, NY, United States; ^8^Department of Bioengineering and Therapeutic Sciences, University of California, San Francisco, San Francisco, CA, United States; ^9^Department of Pediatric Neurology, Cortica Healthcare, San Rafael, CA, United States

**Keywords:** machine-learning, edge density imaging, diffusion tensor imaging, sensory over-responsivity, auditory over-responsivity, neurodevelopmental disorders, sensory processing disorders

## Abstract

Sensory over-responsivity (SOR) commonly involves auditory and/or tactile domains, and can affect children with or without additional neurodevelopmental challenges. In this study, we examined white matter microstructural and connectome correlates of auditory over-responsivity (AOR), analyzing prospectively collected data from 39 boys, aged 8–12 years. In addition to conventional diffusion tensor imaging (DTI) maps – including fractional anisotropy (FA), mean diffusivity (MD), radial diffusivity (RD), and axial diffusivity (AD); we used DTI and high-resolution T1 scans to develop connectome Edge Density (ED) maps. The tract-based spatial statistics was used for voxel-wise comparison of diffusion and ED maps. Then, stepwise penalized logistic regression was applied to identify independent variable (s) predicting AOR, as potential imaging biomarker (s) for AOR. Finally, we compared different combinations of machine learning algorithms (i.e., naïve Bayes, random forest, and support vector machine (SVM) and tract-based DTI/connectome metrics for classification of children with AOR. In direct sensory phenotype assessment, 15 (out of 39) boys exhibited AOR (with or without neurodevelopmental concerns). Voxel-wise analysis demonstrates extensive impairment of white matter microstructural integrity in children with AOR on DTI maps – evidenced by lower FA and higher MD and RD; moreover, there was lower connectome ED in anterior-superior corona radiata, genu and body of corpus callosum. In stepwise logistic regression, the average FA of left superior longitudinal fasciculus (SLF) was the single independent variable distinguishing children with AOR (*p* = 0.007). Subsequently, the left SLF average FA yielded an area under the curve of 0.756 in receiver operating characteristic analysis for prediction of AOR (*p* = 0.008) as a region-of-interest (ROI)-based imaging biomarker. In comparative study of different combinations of machine-learning models and DTI/ED metrics, random forest algorithms using ED had higher accuracy for AOR classification. Our results demonstrate extensive white matter microstructural impairment in children with AOR, with specifically lower connectomic ED in anterior-superior tracts and associated commissural pathways. Also, average FA of left SLF can be applied as ROI-based imaging biomarker for prediction of SOR. Finally, machine-learning models can provide accurate and objective image-based classifiers for identification of children with AOR based on white matter tracts connectome ED.

## Introduction

Sensory over-responsivity (SOR) is a facet of sensory modulation dysfunction characterized by exaggerated, intense, or prolonged behavioral response to sensations not typically perceived as threatening, harmful, or noxious (Schoen et al., 2008). Auditory over-responsivity (AOR), or auditory hypersensitivity, is defined by heightened and atypical reaction to auditory stimuli that are neither threatening nor uncomfortably loud for a typical individual’s perception (Gee et al., 2014). SOR, to both auditory and/or tactile stimuli, is commonly reported in children with anxiety disorders, attention-deficit hyperactivity disorder (ADHD), and autism spectrum disorder (ASD) (Green et al., 2013; Conelea et al., 2014; Ben-Sasson et al., 2017; Tavassoli et al., 2019). In large cohorts of school-aged children (7–10 years of age), SOR – including auditory, tactile, visual, proprioceptive, and vestibular sensory domains – was found in 8–15% of children; while approximately 25–60% of children with SOR also met criteria for a psychiatric disorder (Carter et al., 2011; Van Hulle et al., 2012, 2015; Conelea et al., 2014). Although we have previously shown strong posterior predominant differences in local white matter microstructure in boys and girls with sensory processing disorders (SPD) (Chang et al., 2015; Brandes-Aitken et al., 2018a), we have not yet investigated whether children with AOR (a concise subset of the broader SPD cohort) show differences that are specific to this aspect of sensory processing abnormality, or how this affects whole brain connectivity.

Voxel-wise DTI studies have shown microstructural changes in white matter of children with ASD, SPD, and ADHD (Chang et al., 2014; Aoki et al., 2017). The most commonly studied DTI metrics of white matter integrity are fractional anisotropy (FA), mean diffusivity (MD), radial diffusivity (RD), and axial diffusivity (AD). While these DTI measures are highly sensitive to microstructural changes, they lack specificity and can be affected by demyelination/dysmyelination, axonal diameters, or neural fiber density (Mukherjee et al., 2008a,b). Overall, FA is highly sensitive to microstructural changes, but less specific to the type of change; MD is sensitive to cellularity, edema, and necrosis; AD tends to decrease in axonal injury but increases with brain maturation; whereas, RD increases with de- or dys-myelination (Feldman et al., 2010; Alexander et al., 2011) Recently, edge density imaging (EDI) has been proposed for topographic assessment of connectome edges in cerebral white matter (Owen et al., 2015, 2016; Wang et al., 2017). Although not proven histologically, EDI is theoretically constructed to provide a more specific measure of nerve fiber tracts connecting structural gray matter hubs in the brain. Preliminary data on EDI have shown greater density of connectomic edges in posterior white matter pathways, which are commonly affected in neurodevelopmental disorders (Owen et al., 2015). This finding suggested a role for EDI in assessment of microstructural and connectomic changes in children with sensory-based neurodevelopmental disorders.

While heterogeneous manifestations of sensory processing abnormalities have been recognized and studied in the context of ASD or SPD; in this study we aimed to take a sensory-first approach to understanding the neurobiology of auditory SOR phenotype – focusing on AOR across children with or without additional neurodevelopmental conditions. At the first step, we applied voxel-wise analysis to examine the white matter microstructural, and connectomic correlates of the AOR by examining conventional DTI metrics (i.e., FA, MD, RD, and AD), and connectome EDI, respectively. Then, we applied a stepwise penalized logistic regression model to identify the independent tract-based variable (s) that can distinguish children with AOR from those without. Such tract-based predictors for AOR can be applied as a simple region-of-interest (ROI)-based tool for identification of children with AOR. The penalized logistic regression is optimized for multivariate analysis with high level of collinearity between variables – such as mean FA in adjacent white matter tract. Finally, we applied different supervised machine-learning models for classification of AOR, integrating multitude of tract-based DTI/EDI metrics. Machine-learning algorithms allow relating the input data to output classification based on training cohorts and without being explicitly programmed. However, there is no generalizable rule to determine which algorithm achieve optimal classification in a given dataset, thus requiring training and validation of different models for direct comparison in different datasets. The comparative evaluation in our study aimed to identify the combination of machine-learning algorithm and DTI/EDI metrics with the highest accurate classification rates for AOR; and to demonstrate the feasibility of this methodology for devising new imaging biomarkers for identification of children with AOR based on white matter microstructural and connectomic correlates.

## Materials and Methods

### Subjects

The participants in this study were recruited through our institute Sensory Neurodevelopment and Autism Program research database. A cognitive and behavioral child neurologist examined all children. Only boys aged 8–12 years were included in this study to reduce potential confounding effects of gender and age. Participants were included regardless of the presence or absence of additional neurodevelopment challenges. The AOR cohort assignment was determined using a direct assessment tool – Sensory Processing 3-Dimensions Assessment (SP-3D:A) – conducted by an occupational therapist with research validation from the STAR Institute in Denver, CO (Mulligan et al., 2018; Tavassoli et al., 2019). Some children in our cohort had been additionally categorized in previous research studies as having ASD or SPD. The ASD assignment included a community diagnosis, a score of ≥15 on the Social Communication Questionnaire, and a confirmed ASD classification with the Autism Diagnostic Observation Schedule (Lord et al., 1994, 2000; Chang et al., 2014, 2015). Participants with an SPD designation had been diagnosed by a community occupational therapist and a score in the “Definite Difference” range (<2% probability) in at least one of the Sensory Profile sections (Brandes-Aitken et al., 2018a,b; Tavassoli et al., 2019). The differential screening test for processing (DSTP) was used to evaluate the acoustic and linguistic discrimination of auditory processing module in children using subtests assessing phonic and phonemic manipulation (Demopoulos et al., 2017). The University Institutional Review Board approved this study. Written, informed consents from primary caregivers and assent from study participants were obtained.

### Image Acquisition Protocol

All children were scanned on a 3-Tesla MRI scanner (Siemens, Tim Trio, Erlangen, Germany) using a 12-channel head coil. Whole brain DTI were acquired using a twice-refocused diffusion-weighted echoplanar sequence with Echo Time = 8000 ms; Repetition Time = 109 ms, Field of view = 220 mm; voxel size = 2.2 × 2.2 × 2.2 mm; 64 non-collinear diffusion directions at *B*-value of 2000 s/mm^2^; and one image with no diffusion weighting. We also obtained T1-weighted images using 3-dimensional magnetization-prepared rapid acquisition gradient echo for anatomical registration (Echo Time = 2.98 ms, Repetition Time = 2300 ms, inversion time = 900 ms, flip angle = 9°) (Payabvash et al., 2019).

### DTI Post-processing

All image processing and analyses were conducted using publicly available FSL 5.0.8 software (Oxford, United Kingdom). After eddy current and motion corrections, the non-brain tissue was removed, and the diffusion maps for FA, MD, RD, and AD were developed using the FSL Diffusion Toolbox software (Chang et al., 2014, 2015).

### Edge Density Imaging

The imaging pipeline for development of EDI has been described previously (Owen et al., 2015; Payabvash et al., 2019). The FSL BEDPOSTX tool was used for probabilistic tractography and modeling multiple fiber orientations per each voxel. The BEDPOSTX automatically determines the number of crossing fiber at each voxel; applying the default recommendations from toolbox, the number of fibers modeled per voxel was set to 2, with multiplicative “weight” factor of 1, and 1000 “burn in” iterations. Then, 82 cortical and subcortical regions were automatically segmented on the T1-weighted images using Desikan-Killiany Atlas (Desikan et al., 2006), provided in Freesurfer 5.3.0 (Massachusetts General Hospital, Boston, MA, United States). These regions were then coregistered to diffusion space using a linear affine transformation, serving as nodes of connectome, and employed as seed/target regions for probabilistic tractography using FSL probtrackx2 (Owen et al., 2015). The probabilistic tractography was applied with numbers of samples set to 5000, maximum steps of 2000, step length of 0.5 mm, and curvature threshold of 0.2 (Owen et al., 2015). The total number of estimated tracts (i.e., structural connectome edges connecting the nodes) through each voxel in white matter was calculated as the edge density (ED) value for that voxel (Owen et al., 2015, 2016).

### Tract-Based Spatial Statistics (TBSS)

Tract-based spatial statistics was used for coregistration and voxel-wise comparison of DTI and EDI maps. All FA maps were registered to the most representative map among the cohort, and then onto MNI-152 standard space. The corresponding coregistration matrixes were applied for coregistration of other diffusion maps (i.e., MD, RD, and AD) as well as ED onto the MNI-152 space. A skeletonized image was developed from the mean of all aligned FA maps, and thresholded to exclude voxels with FA < 0.15. The “Randomise” tool from FSL was used for voxel-wise analysis of diffusion and EDI maps, applying 5000 non-parametric permutations, and threshold-free cluster enhancement (TFCE) for family-wise error correction. The age, presence of ASD, and SPD were included as covariates in General linear model (GLM).

### Voxel-Based Morphometry (VBM)

We used the VBM tool in FSL to evaluate voxel-wise differences in focal gray matter volume/topography between two study groups (Smith et al., 2004; Douaud et al., 2007). An isotropic Gaussian kernel with a sigma of 3 mm was applied on modulated gray matter images. The voxel-wise GLM was applied after 5000 permutation-based non-parametric testing, and correcting for multiple comparisons with TFCE.

### Machine-Learning Models

Four different machine-learning models were applied in this study: naïve Bayes, Random forest, support vector machine (SVM) with linear, and polynomial kernels. The average FA, MD, RD, and ED of 48 white matter tracts were calculated and used as input for these models; the average AD variables were not used since there was no significant difference on TBSS voxel-wise analysis. The white matter tracts were based on JHU ICBM-DTI-81 template in FSL, which were warped into each subject’s native diffusion space by inverse spatial transformations estimated from the population-specific template generated in TBSS process.

The statistical “r” packages^1^ were used to devise machine-learning models. These statistical packages are publicly available and specific modifications implemented in each model are detailed below: For naïve Bayes models, we applied a high-performance implementation of the algorithm provided in “naivebayes” package. Naïve Bayes are probabilistic classifiers, calculating the probability of each category – with a (naïve) assumption, that every predictor is independent of the others – and the category with the highest probability will be the model output. Assuming that predictor metrics follow Gaussian distribution, no kernel was applied. The Laplace smoothing was also set to zero. For random forest models, we applied the “randomForest” package for classification and regression based on ensembles of decision trees. Each decision tree is structured as a sequence of questions (splits) for classification of cohort based on the value of one or a series of predictor variables; and a final prediction for the classification is made at terminal nodes (leaves of the tree). In our preliminary experiments with different metrics, error rate plateaued after 160 to 320 trees, so the default implementation of 500 trees per each model seems adequate to achieve the lowest error rate among permutations. As recommended by authors of the package, a randomly selected one-third subset of variables was tried at each split. For SVMs, we applied the “e1071” package with linear and non-linear (i.e., polynomial) kernels. In SVMs, the data points (e.g., subjects) are viewed as multi-dimensional vectors, where the number of dimensions equals the number of variables; and a “hyperplane” is constructed to separate (classify) the data points. While the simplest hyperplanes are linear classifiers; non-linear kernel hyperplanes may potentially achieve better classification. In our preliminary studies, a cost of 0.1 returned the optimal error rate for linear kernel. For polynomial kernels, a sigma of 1 was applied as per the default setting.

Given the small sample size and to reduce the effects of overfitting, we compared the performance of different combinations of machine-learning models with DTI/EDI metrics based on averaged test metrics from cross validation. Therefore, subjects were randomly divided into training and validation samples × 500 times for stratified cross-validation, preserving the ratio of children with AOR to those without in training and validation samples. In each permutation, the machine learning models were trained on the randomly selected training dataset, and tested on corresponding validation sample. Based on predictions in validation sample, a confusion matrix was developed to determine the accuracy, sensitivity, specificity, positive predictive value (PPV), and negative predictive value (NPV). Then, the average (95% confidence interval) of test characteristics among 500 cross validation samples were calculated for different combinations of machine-learning models and DTI/EDI metrics, and presented in heatmap format for comparative assessment.

### Statistics

Children’s age are presented as average ± standard deviations, and compared using student *t*-test; whereas, children’s performance on SP-3D: A cognitive tests are expressed as median (interquartile) and compared using Mann–Whitney *U*-test. For stepwise penalized logistic regression analysis, the tract-based DTI or EDI average values were used as input. We applied the “stepPlr” package with forward and backward stepwise variable selection and 0 maximum interaction. Notably, introduction of any level of interaction between variables resulted in unstable regression model. Receiver operating characteristics analysis was performed to determine the accuracy of independent variable (s) in prediction of AOR. A causal mediation analysis was performed to test whether the correlation of tract-specific microstructural changes and AOR was mediated via auditory-linguistic discrimination abnormalities measured by DSTP – using the “mediation” package in “r”.

## Results

### Subjects’ Characteristics

A total of 39 boys were included in this study. Among these, 15 children (38%) fulfilled the criteria for AOR. There was no significant difference in average age of children with AOR (11.3 ± 1.1 years) versus those without (11.7 ± 1.5 years, *p* = 0.157). In addition, 4/15 (27%) children with AOR and 3/24 (13%) of those without AOR fulfilled the criteria for ASD diagnosis (*p* = 0.396); and 8/15 (53%) children with AOR and 6/24 (25%) of those without AOR fulfilled the criteria for SPD (*p* = 0.095). On clinical assessment, the median SP-3D:A score of children with AOR was 2 (2 – 4) compared to 0 (0 – 1) in children without AOR (*p* < 0.001).

### White Matter Tract Diffusion Tensor and Connectomic Correlates of AOR

**FIGURE 1:**
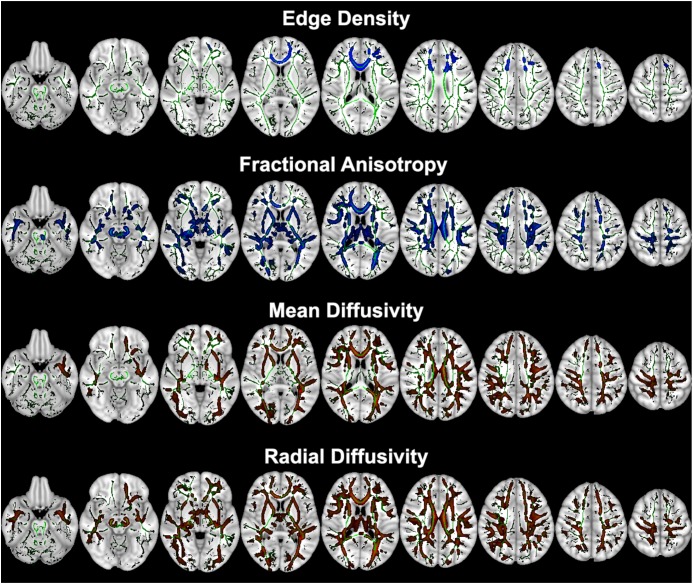
Tract-Based Spatial Statistics (TBSS) voxel-wise analysis of ED and diffusion tensor metrics between children with AOR (*n* = 15) versus those without (*n* = 24). The voxels from white matter tracts with significant difference between the two study groups are overlaid on mean skeletonized FA averaged from all aligned FA maps (green). Children with AOR had lower white matter tract ED and FA (colored blue) but higher MD and RD (colored red) compared to those without. The Supplementary Table S1 lists the white matter tracts with significant voxel-wise difference between two study groups. Of note, images are depicted in radiological view (i.e., left hemisphere on the right). AOR, Auditory over-responsivity; ED, Edge Density; FA, Fractional Anisotropy; MD, Mean Diffusivity; RD, Radial Diffusivity; TBSS, Tract-based spatial statistics.

Comparing 15 children with AOR versus 24 without, the voxel-wise comparisons of DTI metrics revealed that children with AOR had lower voxel-wise FA but higher MD and RD compared to those without AOR throughout anterior and posterior white matter tracts (Figure 1). Children with AOR also had lower ED in anterior and superior corona radiata, genu and body of corpus callosum (Figure 1 and Supplementary Table S1). There was no significant difference in voxel-wise analysis of AD – with the lowest voxel-wise *p*-value of 0.192. The Supplementary Table S1 lists the white matter tracts with significant voxel-wise difference in DTI metrics and ED between the two study groups. Overall, the extent of voxel-wise difference between the two study groups were more extensive on FA, MD, and RD maps compared to ED maps. The patient’s age, presence of ASD and SPD criteria had no significant effect on voxel-wise results using GLM.

**FIGURE 2:**
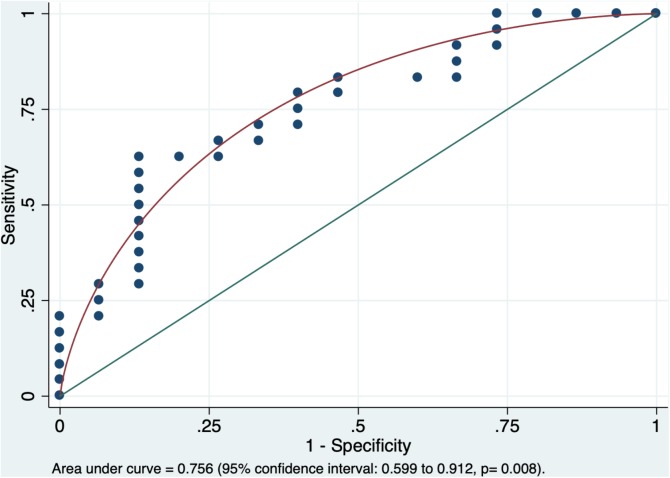
The receiver operating characteristic analysis yielded an area under the curve of 0.756 (95% confidence interval: 0.599–0.912) for average FA of left SLF in prediction of AOR (*p* = 0.008). The average FA of the left SLF was the only independent variable distinguishing children with AOR from those without in the stepwise penalized logistic regression. AOR, Auditory over-responsivity; FA, Fractional Anisotropy; SLF, superior longitudinal fasciculus.

In multivariable stepwise penalized logistic regression model, among all tract-based tensor metrics (i.e., ED, FA, MD, and RD), the single independent variable distinguishing children with AOR from those without was the left superior longitudinal fasciculus (SLF) average FA (*p* = 0.007). The area under the curve in receiver operating characteristic analysis for prediction of AOR based on the left SLF average FA was 0.756 (95% confidence interval: 0.599 to 0.912, *p* = 0.008, Figure 2). Given the consideration that the SLF has been previously associated with auditory discrimination and ASD label, the mediation analysis was preformed using DSTP direct assessment. However, there was no significant mediated effect from DSTP auditory (*p* = 0.44) or linguistic (*p* = 0.64) scores on correlation of the left SLF average FA with presence of AOR.

### Machine-Learning Analysis for Identification of AOR

**FIGURE 3:**
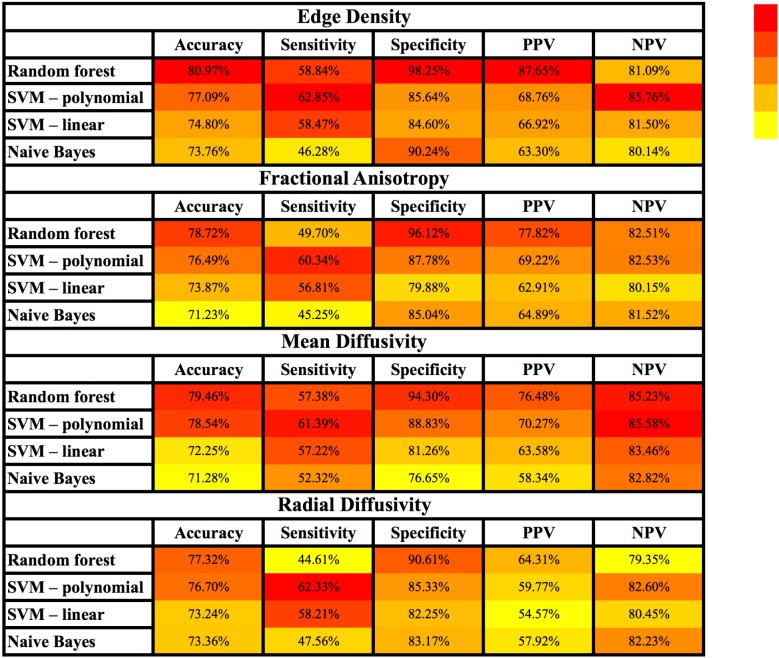
Heatmap depiction of different supervised machine-learning models applied for classification of children with AOR. Of note, the color tone is applied for each column separately to facilitate visual comparison of each test characteristic (e.g., sensitivity) among different models. Each cell represents the average performance of corresponding machine-learning algorithm among × 500 stratified randomly selected validation samples (details in Supplementary Table S2). Among different combinations of machine learning models and connectivity metrics, the combination of “Edge Density” with random forest models yields the highest accuracy, specificity and PPV; whereas polynomial SVM yielded the highest sensitivity and NPV. AOR, Auditory over-responsivity; NPV, negative predictive value; PPV, positive predictive value; SVM, Support Vector Machine.

Figure 3 and Supplementary Table S2 summarize the results of different machine-learning algorithms applied for classification of children with AOR. Overall, the models using tract-based ED had greater accuracy, sensitivity, specificity, PPV, and NPV, when compared to those based on FA, MD, and RD. With regards to different machine-learning methods, ED-based random forest models had greater accuracy, specificity, and PPV compared to other models; whereas, SVM models with polynomial Kernel had higher sensitivity and NPV in identification of children with AOR (Figure 3 and Supplementary Table S2).

### Gray Matter Macrostructural Analysis

Using VBM, there was no significant voxel-wise difference in gray matter regional volume or morphometry between children with and without AOR. Inclusion of patients’ age, ASD, and SPD traits as covariates also showed no significant difference in results of GLM constructs.

## Discussion

Children labeled with ASD, ADHD, or SPD, tend to present with wide-ranging and heterogeneous phenotypes including sensory processing hyper or hypo responsivity. There is a growing interest in SOR, which occurs in children with a range of neurodevelopmental challenges, including attention, anxiety, social, and language disorders. This study is the first study of its kind to investigate the connectomics; and apply machine-learning models for prediction of AOR. The voxel-vise analysis in our study reveals diffuse impairment of the white matter tract integrity (lower FA and higher MD and RD) with cascading effects on connectivity of anterior-superior and inter-hemispheric hub regions (lower ED). While extensive nature of white matter microstructural changes can make it challenging to identify specific imaging biomarker for prediction of AOR, the penalized regression suggests that a ROI-based assessment of the left SLF average FA may provide an easy-to-apply imaging biomarker for prediction of AOR. Finally, we have shown how machine-learning models allow integrating a multitude of topographic connectomic variables for accurate classification of AOR. Among different combinations of machine-learning algorithms and DTI/EDI metrics, the random forest models using tract-based ED yielded greater accuracy in identification of children with AOR. These preliminary results offer more insight to underlying neural network correlates of AOR, propose average FA of left SLF as an imaging biomarker for AOR, and open a new horizon in application of machine-learning models for devising novel imaging biomarkers.

Extensive white matter microstructural impairment in children with AOR was reflected by reduced FA and elevated MD and RD in anterior and posterior white matter tracts as well as commissural tracts of the corpus callosum on voxel-wise analysis (Figure 1 and Supplementary Table S1). These changes in DTI metrics represent impaired white matter microstructural integrity, and may be due to thinner axon diameter, lower axonal density, or impaired myelin integrity (Mukherjee et al., 2008a,b). Children with ASD and ADHD have also demonstrated extensive microstructural impairments in DTI studies, which may represent shared pathways in children with similar phenotypic presentation (e.g., AOR) (Ameis et al., 2016). On the hand, some studies have reported distinctive patterns in children with ASD, such as impaired connectivity in temporal tracts, which are related to social-emotional processing (Chang et al., 2014). Another study has recently shown impaired connectivity with reduced ED in the posterior white matter and splenium of corpus callosum in children with ASD (Payabvash et al., 2019). While the criteria for ASD, SPD, or ADHD tend to cover a broad spectrum of symptoms, and classify heterogeneous groups of children under the same category, the methodology used in current study paves the road for devising objective and quantitative biomarkers for distinction of children with specific shared phenotype – i.e., AOR.

In addition to extensive impairment of white matter connectivity, we found lower ED in anterior (and to lesser extent superior) corona radiata as well as the genu and body of corpus callosum. While conventional DTI metrics – such as FA, MD, and RD – provide measures of water diffusivity (and therefore white matter microstructural integrity) with no attention to directionality of potential connecting tracts passing through each voxel, the ED specifically measures the number of potential tracts connecting pre-determined gray matter hub at each voxel (Owen et al., 2015). Our findings, theoretically suggest that in children with AOR, the microstructural impairment in anterior-superior white matter pathways and corresponding commissural fibers through corpus callosum may be due to reduced density of neural fibers (reflected by connectome edges); whereas DTI metrics changes in posterior white matter pathways may reflect axonal disorganization without significant reduction in neural fiber density. Notably, the impairment of white matter microstructural integrity and reduced density of connectomic edges in anterior tract pathways were not associated with significant changes in gray matter morphology and volume according to VBM results. Nevertheless, the precise neurohistological correlates of these imaging findings remain to be established.

We also applied stepwise penalized logistic regression to identify the independent variable (s) distinguishing children with AOR from those without. The penalized logistic regression, which gained popularity in evaluation of gene-gene and gene-environment interactions (Park and Hastie, 2008), tends to provide a reliable solution for multivariate analysis with variable multicollinearity in small to moderate samples (Shen and Gao, 2008), like DTI metrics of adjacent white matter tracts. In our analysis, the single independent DTI/EDI variable distinguishing children with AOR was the average FA of left SLF. These findings can provide an easy-to-apply tool for neuroradiologists to predict AOR using a ROI-based measurement of the left SLF average FA (Figure 2). These results may also highlight a potential role of left SLF pathway in pathogenesis of AOR.

The SLF is implicated in cognitive functions, such as attention, language, and fine-motor ability (Urger et al., 2015). The left SLF represents one of the most replicated white matter tracts with impaired microstructure in DTI studies of children with ASD (Aoki et al., 2017; Blanken et al., 2017). Higher MD in the temporal portion of left SLF is associated with language impairment in children with ASD (Nagae et al., 2012). Notably, our mediation analysis shows that the effects of left SLF microstructural impairment on AOR were not mediated via the acoustic-linguistic discrimination abnormality on DSTP, raising the possibility that left SLF average FA may represent a hallmark of more pervasive microstructural impairment in AOR as manifested in voxel-wise analysis. The fact that microstructural impairment of the left SLF was the most distinctive pattern in children with AOR may represent an underlying shared mechanism in a portion of children labeled with ASD. This would be an important next step for more specific brain-behavior assessment in neuroimaging research.

Prior neuroimaging studies on children with SOR have applied fMRI for evaluation of functional brain connectivity. These fMRI studies in ASD children with SOR demonstrated over-reactive responses and decreased habituation to mildly aversive sensory stimuli in primary sensory processing regions (Green et al., 2013). In addition, the ASD children with SOR had aberrant modulation of connectivity between pulvinar, sensory-motor and prefrontal cortex during sensory stimulation (Green et al., 2017a). These fMRI findings in ASD children correlated with severity of SOR symptoms (Green et al., 2013, 2016, 2017a,b). Our tensor imaging study add to prior functional connectivity results, and suggest extensive impairment of white matter tracts microstructure and reduced ED in anterior white matter pathways as potential underlying mechanism of defective functional connectivity in children with SOR.

Increased computational power and accumulation of big data have led to much progress in application of machine-learning algorithms in bioimaging over the past decade. The machine-learning models are particularly suitable statistical solutions in construction of classifiers based on multitude of imaging (and clinical) data. However, the “black-box” nature of the machine-learning algorithms imposes a challenge in interpretation of models’ inner workings and decision-making processes. In addition, given that accuracy of machine-learning models generally relies on training with large amount of data points, relatively small size of study cohort raises the possibility of overfitting. Direct comparison of different machine-learning models is also challenging since every time an algorithm is trained on a given dataset, the resultant model can be different; so, it is likely that in a set of training/validation samples, a given model achieves higher accuracy compared to another (say model A performed better than model B), but repeating the training process on the same set of samples yield reverse results (model B performs better than model A). Given these challenges, in this study, we opted to report and compare the averaged results of × 500 cross validation instead of a single model to reduce the possibility of overfitting and provide a realistic estimate of each algorithm accuracy.

The comparative evaluation of four different supervised machine-learning algorithms in our cohort showed that the random forest models achieved greater accuracy, specificity, and PPV compared to others for classification of AOR; whereas, SVM models, especially those applying polynomial kernel, were more sensitive (Figure 3 and Supplementary Table S2). In addition, models using white matter tract ED were more accurate compared to those using FA, MD, or RD. Given that machine-learning algorithms apply different statistical models and mathematical assumptions for classification, it seems pertinent to compare and choose the appropriate algorithm for each clinical setting. One can also suggest that calculating the results of machine-learning algorithms with different sensitivity and specificity may provide a choice for clinicians in their practice to implement quantitative and objective neuroimaging-based results based on their clinical judgment in case by case basis. Furthermore, the application of machine-learning algorithms for development of neuroimaging biomarkers for AOR seems appealing given that the microstructural changes of white matter tracts are not visually perceivable on standard clinical assessment, and are relatively diffuse and symmetrical.

Small sample size and unequal study groups limit the overall power of our study. Moreover, while limiting the inclusion criteria to boys aged 8 to 12 years reduces the confounding effect of gender and age, it also limits the generalizability of results. Finally, there is currently no universal agreed upon assessment for categorizing AOR, although this is a topic of much discussion and development, there remains variability in assessment and classification of these children across clinics and research laboratories.

## Conclusion

The voxel-wise analysis of DTI metrics revealed extensive impairment of white matter tract microstructure among children presenting with AOR phenotype – with or without additional neurodevelopmental disorder criteria. Evaluation of white matter connectome reveals reduced ED in anterior-superior white matter pathways and associated commissural tracts of corpus callosum. In addition, microstructural impairment of the left SLF was the most distinctive variable distinguishing children with AOR from those without, which provides an easy-to-apply ROI-based metric for identification of AOR, and may point out to the fundamental role of this white matter tract in underlying mechanism of AOR. Finally, machine-learning algorithms using tract-based information from tensor imaging and connectomic studies can be applied to devise novel neuroimaging biomarkers for classification of children with SOR. In our cohort, the combination of random forest models using EDI metrics had greater accuracy compared to other machine-learning algorithms and tensor imaging metrics. Such neuroimaging biomarkers can potentially help clinicians with accurate and timely identification of SOR, distinguishing children with SOR trait among those affected by ASD or ADHD, and improving treatment triage/planning.

## Author Contributions

SP, PM, and EM designed the study, interpreted the results, and drafted the manuscript. SP, EP, JO, and MW designed the images analysis method, and contributed to image processing. TT, MG, AB-A, and EM gathered the clinical information and were involved in subject recruitment. All authors reviewed the manuscript and provided critical contribution.

## Conflict of Interest Statement

The authors declare that the research was conducted in the absence of any commercial or financial relationships that could be construed as a potential conflict of interest.

## Footnotes

1 https://www.r-project.org/
