# Supplementary material for: White Matter Connectome Correlates of Auditory Over-Responsivity: Edge Density Imaging and Machine-Learning Classifiers
Source: Front Integr Neurosci. 2019 Mar 29;13:10. doi: 10.3389/fnint.2019.00010 (PMC6450221; doi:10.3389/fnint.2019.00010)
Supplement: Supplementary file 1 [file Data_Sheet_1.docx]

Supplemental Table 1. White matter tracts with significantly different ED and diffusion tensor metrics between children with AOR and those without in voxel-wise analysis.

|  | **ED** | **FA** | **MD** | **RD** |
| --- | --- | --- | --- | --- |
| **Left anterior corona radiata** | 52 | 198 | 267 | 296 |
| **Right anterior corona radiata** | 65 | 235 | 231 | 272 |
| **Left superior corona radiata** | 8 | 202 | 137 | 234 |
| **Right superior corona radiata** | 5 | 234 | 41 | 122 |
| **Left posterior corona radiata** | 0 | 147 | 35 | 117 |
| **Right posterior corona radiata** | 0 | 5 | 0 | 0 |
| **Left anterior limb of internal capsule** | 0 | 146 | 123 | 155 |
| **Right anterior limb of internal capsule** | 0 | 208 | 135 | 189 |
| **Left posterior limb of internal capsule** | 0 | 166 | 48 | 147 |
| **Right posterior limb of internal capsule** | 0 | 196 | 7 | 62 |
| **Left retrolenticular part of internal capsule** | 0 | 132 | 113 | 166 |
| **Right retrolenticular part of internal capsule** | 0 | 191 | 91 | 158 |
| **Left posterior thalamic radiation** | 0 | 281 | 271 | 315 |
| **Right posterior thalamic radiation** | 0 | 239 | 172 | 235 |
| **Left superior fronto-occipital fasciculus** | 0 | 2 | 1 | 2 |
| **Right superior fronto-occipital fasciculus** | 0 | 20 | 25 | 28 |
| **Left superior longitudinal fasciculus** | 0 | 334 | 329 | 380 |
| **Right superior longitudinal fasciculus** | 0 | 334 | 204 | 373 |
| **Genu of corpus callosum** | 435 | 628 | 470 | 629 |
| **Body of corpus callosum** | 253 | 1065 | 658 | 955 |
| **Splenium of corpus callosum** | 0 | 314 | 378 | 429 |
| **Left cingulum** | 0 | 14 | 4 | 5 |
| **Right cingulum** | 0 | 17 | 6 | 13 |
| **Left external capsule** | 0 | 161 | 344 | 338 |
| **Right external capsule** | 0 | 167 | 212 | 236 |
| **Fornix midline** | 0 | 20 | 40 | 56 |
| **Left fornix** | 0 | 90 | 12 | 69 |
| **Right fornix** | 0 | 28 | 0 | 30 |
| **Left sagittal stratum** | 0 | 69 | 50 | 42 |
| **Right sagittal stratum** | 0 | 85 | 73 | 95 |
| **Left tapetum** | 0 | 0 | 12 | 3 |
| **Right tapetum** | 0 | 35 | 35 | 42 |
| **Left uncinate fasciculus** | 0 | 9 | 15 | 17 |
| **Right uncinate fasciculus** | 0 | 14 | 0 | 9 |
| **Left cerebral peduncle** | 0 | 199 | 2 | 163 |
| **Right cerebral peduncle** | 0 | 177 | 0 | 118 |
| **Pontine crossing tract** | 0 | 63 | 74 | 98 |

Each cell represents the number of voxels with significantly different ED or diffusion tensor metrics (p<0.05) between children with and without AOR on voxel-wise TBSS analysis after applying TFCE correction (Figure 1). The number of voxels is extrapolated from projection of voxel-wise results onto the 182×218×182 standard 1-mm MNI-152 brain space. Children with AOR had significantly lower ED and FA, but higher MD and RD compared to those without.

AOR= Auditory over-responsivity; ED= Edge Density; FA= Fractional Anisotropy; MD= Mean Diffusivity; RD= Radial Diffusivity; TBSS= Tract-based spatial statistics; TFCE= threshold-free cluster enhancement

Supplemental Table 2. Test characteristics of different machine-learning algorithms for classification of AOR using tract-based diffusion and connectome metrics.

| **Edge Density** | | | | | |
| --- | --- | --- | --- | --- | --- |
|  | **Accuracy** | **Sensitivity** | **Specificity** | **PPV** | **NPV** |
| **Random forest** | 80.97%  (79.76% – 82.17%) | 58.84%  (56.15% – 61.52%) | 98.25%  (97.17% – 99.33%) | 87.65%  (84.26% – 91.04%) | 81.09%  (80.12% – 82.06%) |
| **SVM – polynomial** | 77.09%  (75.95% – 78.14%) | 62.85%  (61.71% – 63.93%) | 85.64%  (84.33% – 86.98%) | 68.76%  (66.98% – 70.43%) | 85.76%  (84.37% – 86.98%) |
| **SVM – linear** | 74.80%  (73.51% – 76.09%) | 58.47%  (56.12% – 60.81%) | 84.60%  (82.80% – 86.40%) | 66.92%  (64.43% – 69.41%) | 81.50%  (80.33% – 82.68%) |
| **Naive Bayes** | 73.76%  (72.40% – 75.11%) | 46.28%  (43.40% – 49.17%) | 90.24%  (88.48% – 92.00%) | 63.30%  (60.08% – 66.52%) | 80.14%  (78.91% – 81.36%) |
| **Fractional Anisotropy** | | | | | |
|  | **Accuracy** | **Sensitivity** | **Specificity** | **PPV** | **NPV** |
| **Random forest** | 78.72%  (77.38% – 80.05%) | 49.70%  (46.99% – 52.41%) | 96.12%  (94.39% – 97.86%) | 77.82%  (74.83% – 80.81%) | 82.51%  (81.37% – 83.65%) |
| **SVM – polynomial** | 76.49%  (71.14% – 77.84%) | 60.34%  (57.88% – 62.80%) | 87.78%  (85.89% – 89.67%) | 69.22%  (66.60% – 71.83%) | 82.53%  (81.29% – 83.77%) |
| **SVM – linear** | 73.87%  (72.40% – 75.34%) | 56.81%  (54.49% – 59.13%) | 79.88%  (78.16% – 81.59%) | 62.91%  (60.48% – 65.35%) | 80.15%  (79.01% – 81.29%) |
| **Naive Bayes** | 71.23%  (69.94% – 72.52%) | 45.25%  (42.58% – 47.93%) | 85.04%  (83.00% – 87.09%) | 64.89%  (62.05% – 67.73%) | 81.52%  (80.17% – 82.86%) |
| **Mean Diffusivity** | | | | | |
|  | **Accuracy** | **Sensitivity** | **Specificity** | **PPV** | **NPV** |
| **Random forest** | 79.46%  (78.23% – 80.54%) | 57.38%  (54.69% – 60.07%) | 94.30%  (92.65% – 95.95%) | 76.48%  (73.62% – 79.33%) | 85.23%  (84.04% – 86.41%) |
| **SVM – polynomial** | 78.54%  (77.19% – 79.89%) | 61.39%  (58.93% – 63.85%) | 88.83%  (86.94% – 90.72%) | 70.27%  (67.65% – 72.88%) | 85.58%  (84.34% – 86.82%) |
| **SVM – linear** | 72.25%  (70.90% – 73.59%) | 57.22%  (54.81% – 59.64%) | 81.26%  (79.47% – 83.05%) | 63.58%  (61.05% – 66.12%) | 83.46%  (72.27% – 84.65%) |
| **Naive Bayes** | 71.28%  (69.81% – 72.75%) | 52.32%  (49.55% – 55.09%) | 76.65%  (74.69% – 78.62%) | 58.34%  (55.96% – 60.73%) | 82.82%  (81.27% – 84.37%) |
| **Radial Diffusivity** | | | | | |
|  | **Accuracy** | **Sensitivity** | **Specificity** | **PPV** | **NPV** |
| **Random forest** | 77.32%  (75.81% – 78.83%) | 44.61%  (41.74% – 47.49%) | 90.61%  (88.69% – 92.52%) | 64.31%  (61.01% – 67.62%) | 79.35%  (78.11% – 80.59%) |
| **SVM – polynomial** | 76.70%  (75.57% – 77.84%) | 62.33%  (60.27% – 64.40%) | 85.33%  (83.75% – 86.91%) | 59.77%  (57.58% – 61.96%) | 82.60%  (81.57% – 83.64%) |
| **SVM – linear** | 73.24%  (71.89% – 74.58%) | 58.21%  (55.80% – 60.63%) | 82.25%  (80.46% – 84.04%) | 54.57%  (52.04% – 57.11%) | 80.45%  (79.26% – 81.64%) |
| **Naive Bayes** | 73.36%  (71.94% – 74.78%) | 47.56%  (44.77% – 50.36%) | 83.17%  (81.25% – 85.10%) | 57.92%  (55.42% – 60.43%) | 82.23%  (80.72% – 83.73%) |

Detailed results of different supervised machine-learning models for classification of children with AOR. The results are the average (95% confidence interval) performance for each algorithm among ×500 stratified randomly selected validation samples, preserving the ratio of children with and without AOR.

AOR= Auditory over-responsivity; NPV= negative predictive value; PPV= positive predictive value; SVM= Support Vector Machine
